# Accuracy of the “Surprise Question” in Predicting Long-Term Mortality Among Older Patients Admitted to the Emergency Department: Comparison Between Emergency Physicians and Nurses in a Multicenter Longitudinal Study

**DOI:** 10.1089/pmr.2024.0010

**Published:** 2024-08-26

**Authors:** Alexandra Coulon, Delphine Bourmorck, Françoise Steenebruggen, Laurent Knoops, Isabelle De Brauwer, On behalf of the COPE Project

**Affiliations:** ^1^ Palliative Care Unit, Cliniques universitaires Saint-Luc, Brussels, Belgium.; ^2^ Institute of Health and Society, UCLouvain, Brussels, Belgium.; ^3^ Department of Emergency Medicine, Cliniques universitaires Saint-Luc, Brussels, Belgium.; ^4^ Department of Geriatric Medicine, Cliniques universitaires Saint-Luc, Brussels, Belgium.

**Keywords:** emergency department (ED), older patients (OP), palliative care (PC), surprise question (SQ), 12-month mortality, Emergency physician (EP), Emergency nurse (EN)

## Abstract

**Background:**

The “surprise question” (SQ) (“Would you be surprised if this patient died in the next 12 months?”) is the most frequently used screening tool in emergency departments (EDs) to identify patients with poor prognosis and potential unmet palliative needs.

**Objective:**

To test and compare the accuracy of the SQ between emergency nurses (ENs) and emergency physicians (EPs) in predicting long-term mortality among older patients (OP) in the ED.

**Design and Setting/Subjects:**

A prospective cohort study of OPs (≥75 years) conducted in two Belgian EDs. EPs and ENs answered the SQ for the patients they cared for. Positive SQ (SQ+) was defined as a “no” answer. One-year mortality was assessed by phone call.

**Results:**

EPs and ENs both answered the SQ for 291 OPs (mean age 83.2 ± 5.4, males 42.6%). The SQ was positive in 43% and 40.6%, respectively. Predictive values were similar in both groups: sensitivity, specificity, c-statistics, negative predictive value, and positive predictive value were 0.79 (0.66–0.88), 0.68 (0.62–0.76), 0.69 (0.63–0.75), 0.92 (0.86–0.96), and 0.4 (0.31–0.50), respectively, for EPs and 0.71 (0.57–0.82), 0.69 (0.62–0.75), 0.69 (0.63–0.75), 0.89 (0.83–0.93), and 0.41 (0.31–0.51), respectively, for ENs. SQ + was associated with a higher mortality risk in both group (EPs hazard ratio: 3.2 [1.6–6.7], *p* = 0.002; ENs hazard ratio: 2.5 [1.3–4.8], *p* = 0.006). The survival probability was lower when both EPs and ENs agreed on the SQ+ (*p* < 0.001).

**Conclusion:**

The SQ is a simple tool to identify older ED patients at high mortality risk. Concordant responses from EPs and ENs are more predictive than either alone.

## Introduction

The global population is aging, leading to a rise in the number of older patients (OP) facing one or more progressive chronic conditions and frailty.^1^ Insufficient consideration is given to discussions related to the patients’ goals of care and end-of-life issues.^2,3^ Palliative care (PC) is often introduced at a very late stage, despite well-established benefits when applied earlier.^4^ Such a PC approach involves anticipating end-of-life issues and patient’s wishes, while sometimes maintaining certain treatments. This approach facilitates a gradual transition to terminal phases.

Acute exacerbations of the diseases often lead to a nonscheduled hospital admission, through the emergency department (ED). Such admission is commonly seen as an adverse event,^5^ indicating an acute decline in health or the progression of the disease.^6,7^ However, it can also be used as an opportunity to identify OPs who could benefit from PC and to assess potentially unmet needs.^8–10^ Benefits of initiating early PC in the ED have been proven in terms of costs and length of hospital stay.^11^

Various tools have been suggested in the literature to facilitate the identification of these OPs.^12,13^ In the fast-paced and crowded environment of the ED, it is essential for such a tool to be quick and user-friendly.^13,14^

The “surprise question” (SQ) is the most commonly used prognostic tool in the ED, whereby clinicians are asked “Would you be surprised if this patient died in the next 6–12 months?”^13^ The SQ has proven its value in facilitating discussions about PC and end-of-life considerations.^15^

Two systematic reviews with meta-analysis^16,17^ and one systematic review^13^ examined the accuracy of the SQ in predicting mortality, with widely varying results (from 11.6% to 95.6%). Although it has been tested in various settings such as oncology, hemodialysis, and surgery,^18–20^ there is a limited number of studies in EDs. This tool has demonstrated its feasibility, usefulness, and quickness when applied in this setting.^15^ Recent research has explored the accuracy of the SQ in this context,^21^ focusing on predicting short-term mortality.^22,23^ Moreover, one study highlighted a higher accuracy of the SQ in predicting 30-days mortality of OPs in the ED when emergency nurses (ENs) and emergency physicians (EPs) agreed.^24^ To date, no study has been carried out in the context of EDs to assess the level of agreement between physicians and nurses in their responses to the SQ in predicting one-year mortality.

Our study sought to investigate (1) the prognostic value of the SQ among physicians and nurses and (2) the potential agreement in responses to the SQ between them.

**Research aims and hypotheses:** We hypothesized that the prognostic value of the SQ from EPs and ENs in predicting long-term mortality would be moderate (accuracy between 0.7 and 0.9^25^) and correlated with ENs’ SQ answers. Moreover, we hypothesized that independent SQ agreement between EPs and ENs improves the SQ accuracy.

## Methods

The study was reported following the STROBE statement.

### Study design and population

This study was a secondary analysis of a bicenter cohort enrolled between December 2019 and November 2020, with interruption due to the COVID-19 pandemic for security reasons (between February and August).

Given the definition of the Belgian care program for the geriatric patient, we included patients aged 75 years and over.^26^ Patients were also eligible if they had been admitted to the ED of two tertiary hospitals in Belgium. Dying patients, i.e., likely to die within 24 hours following ED admission, and patients previously included in the study were excluded. Dying patients were prospectively determined by the researcher after discussion with the EPs in charge.

### Data collection and measurements

During the study period, the following data were collected by three researchers on working days (weekdays from 8 am to 6 pm) from patients or their legal representatives: sociodemographic characteristics, including age, gender, residence, and marital status, Charlson Comorbidity Index (CCI),^27^and prehospital (2 weeks before admission) functional independence as assessed by the basic activities of daily living (bADL, Katz score^28^) and instrumental activities of daily living (iADL, Lawton score^29^) tools.^31^ The CCI is a scoring system used in research to predict outcomes (functional decline, mortality, and length of hospital stay). The number and severity of comorbidities are measured from a predefined list of 19 comorbidities. The Katz and Lawton scores both assess patient’s autonomy. Katz score includes 6 domains rated from 1 to 4, whereas the Lawton score assesses 7 domains. Lower score for the bADL and higher scores for iADL indicate greater autonomy.

During the patient’s ED stay, EPs and ENs answered independently the SQ, i.e., “Would you be surprised if this patient died in the next 12 months?” The researchers asked orally the SQ to the ENs and EPs in charge of the patient, when they had enough clinical information. It was not integrated in the EMR. Physicians were classified according to their length of practice as junior (≤3 years) or senior (>3 years). There were three possible answers: “yes” (“surprised” corresponding to a negative SQ or “SQ-”), “no” (“not surprised” corresponding to a positive SQ or “SQ+”), and “don’t know.” Nurses and physicians were blinded to each other responses.

### Outcomes

The primary outcome was 1-year mortality of OPs admitted to the ED, which was assessed by phone calls to them or their legal representatives.

### Statistical analysis

Descriptive statistics were conducted to analyze the study population. Categorical variables were expressed as numbers and percentages, whereas continuous variables were presented as means and standard deviations. Logistic multiple regression was used to explore potential associations between some parameters and a SQ+. Pearson’s Chi-squared test with Yates’ continuity correction was used to examine the association between EPs’ and ENs’ responses. The agreement between EPs’ and ENs’ responses was evaluated using Cohen’s kappa coefficient.^30^

To evaluate the accuracy of the SQ in predicting 1-year mortality, areas under the receiver operating characteristic (ROC) curves were calculated. ROC curves were generated for each group, and sensitivity and specificity were determined using a bivariate model. Additional metrics such as c-statistics, predictive values, and likelihood ratios were also computed. The performance measures evaluated included sensitivity, specificity, positive and negative predictive values (PPV and NPV), as well as positive and negative likelihood ratios (PLR and NLR). We also examined the agreement between EPs and ENs on their responses to the SQ. Furthermore, the ROC curves of both SQ responses (EPs as Predictor 1 and ENs as Predictor 2) were compared using a test described by Delong et al. for paired ROC curves.^31^

Survival analyses were conducted using Kaplan–Meier curves, and multiple Cox regressions were performed with adjustments for age, gender, and the CCI. These analyses aimed to assess the correlation between SQ responses and 1-year mortality. The Benjamini–Hochberg false-discovery rate correction method (correction for multiple testing) was used to show a statistically significative difference between the survival curves.

Statistical analyses were performed using the R statistical software version 4.3.0 (R packages: “epiR” version 2.0.60, “survival” version 3.5.5, “pROC” version 1.18.2), with statistical significance set at *p* < 0.05.

### Ethics

This study was approved by the medical ethics committee of the two tertiary hospitals and is registered under the number B403201941609. Each participant, or their legal representative, signed an informed consent before any data collection.

## Results

### Population characteristics and 1-year mortality

**FIG. 1.**
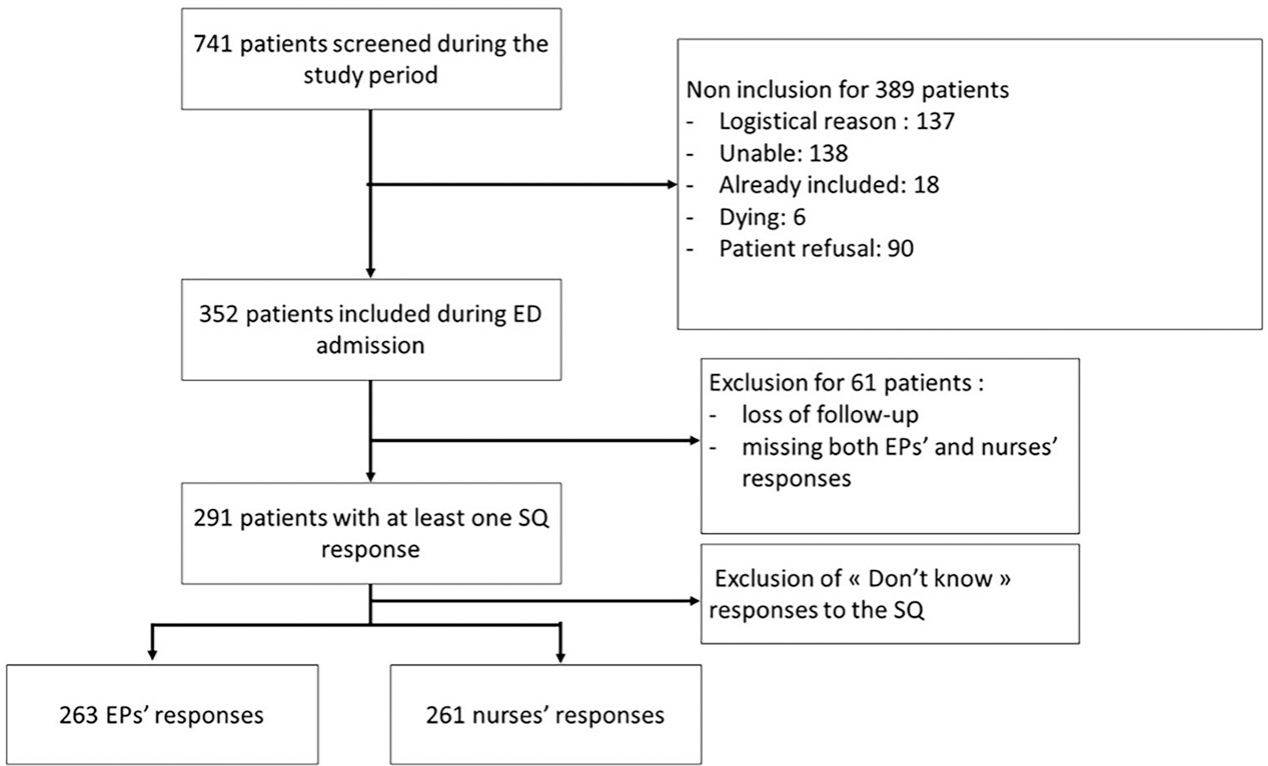
Flowchart of patients’ inclusion.

Figure 1 summarizes the inclusion process from which the final cohort for analysis comprising 291 patients was derived.

Table 1 and Table 2 provide an overview of the characteristics of the 291 patients included, after excluding patients due to loss of follow-up, patients who were unabled to answer or missing of both EPs’ and ENs’ SQ responses. Logistical reasons were the unavailability of the researcher for the patients’ inclusion.

**Table 1.** Patients’ Characteristics and Emergency Physicians’ Responses to the Surprise Question

	Total (n patients = 291) Mean (SD) or n (%)	Nurses (n patients = 263)		*p* value
		SQ+ (*n* = 113) Mean (SD) or n (%)	SQ-(*n* = 150) Mean (SD) or n (%)	
Age, years (SD)	83.2 (5.4)	84.2 (5.3)	82.3 (5.4)	0.006^*^
85+, n (%)	118 (40.5)	55 (48.7)	51 (34)	0.03^*^
Gender, male, n (%)	124 (42.6)	55 (48.7)	56 (37.3)	0.07
Residence, n (%)				
Community-dwelling	267 (91.8)	99 (87.6)	142 (94.7)	0.05
Nursing home	18 (6.2)	12 (10.6)	5 (3.3)	0.02^*^
Partnership	146 (50.2)	56 (49.6)	78 (52)	0.8
CCI, n (SD)	5.9 (2.3)	6.6 (2.5)	5.4 (1.9)	<0.001^*^
Medications, n (SD)	7 (4)	8 (4)	6 (4)	0.005^*^
bADL (Katz), total, n (SD)	8.4 (3.5)	9.5 (4.2)	7.6 (2.6)	<0.001^*^
iADL (Lawton), total, n (SD)	4.6 (2.1)	3.8 (2.2)	5.2 (1.8)	<0.001^*^
Falls (any during the past 12 months), n (%)	131 (45)	56 (49.6)	62 (41.3)	0.23
Death, 12 months, n (%)	63 (21.6)	44 (38.9)	12 (8)	<0.001^*^
Hospitalization, n (%)				
Geriatrics	39 (13.4)	20 (17.7)	13 (8.7)	0.9
Medicine	64 (22)	36 (31.9)	23 (15.3)	0.9
Surgery	17 (5.8)	8 (7.1)	6 (4)	0.9
IMC stroke unit	7 (2.4)	3 (2.7)	3 (2)	0.9
ICU	8 (2.7)	6 (5.3)	2 (1.3)	0.9

bADL, basic activities of daily living; CCI, Charlson comorbidity index; iADL, instrumental activities of daily living; ICU, intensive care unit; IMC, intermediate care; SD, standard deviation; SQ, surprise question.

^*^ Statistically significant

**Table 2.** Patients’ Characteristics and Emergency Nurses’ Responses to the Surprise Question

	Total (n patients = 291) Mean (SD) or n (%)	Nurses (n patients = 261)		*p* value
		SQ+ (*n* = 105) Mean (SD) or n (%)	SQ-(*n* = 156) Mean (SD) or n (%)	
Age, years (SD)	83.2 (5.4)	84.2 (5.6)	82.5 (5.1)	0.01^*^
85+, n (%)	118 (40.5)	49 (46.2)	57 (36.8)	0.16
Gender, male, n (%)	124 (42.6)	51 (48.1)	61 (39.4)	0.16
Residence, n (%)				
Community-dwelling	267 (91.8)	93 (87.7)	145 (93.5)	0.12
Nursing home	18 (6.2)	11 (10.4)	6 (3.9)	0.05
Partnership	146 (50.2)	49 (46.2)	77 (49.7)	0.69
CCI, n (SD)	5.9 (2.3)	6.9 (2.8)	5.4 (1.9)	<0.001^*^
Medications, n (SD)	7 (4)	7.6 (4)	6.5 (3.9)	0.03^*^
bADL (Katz), total, n (SD)	8.4 (3.5)	9.8 (4.5)	7.7 (2.5)	<0.001^*^
iADL (Lawton), total, n (SD)	4.6 (2.1)	3.8 (2.3)	5 (1.9)	<0.001^*^
Falls (any during the past 12 months), n (%)	131 (45)	55 (51.9)	63 (40.6)	0.09
Death, 12 months, n (%)	63 (21.6)	41 (38.7)	17 (11)	<0.001^*^
Hospitalization, n (%)				
Geriatrics	39 (13.4)	19 (17.9)	16 (10.3)	0.16
Medicine	64 (22)	36 (34)	20 (12.9)	0.16
Surgery	17 (5.8)	5 (4.7)	11 (7.1)	0.15
IMC stroke unit	7 (2.4)	2 (1.9)	4 (2.6)	0.15
ICU	8 (2.7)	4 (3.8)	3 (1.9)	0.16

bADL, basic activities of daily living; CCI, Charlson comorbidity index; iADL, instrumental activities of daily living; ICU, intensive care unit; IMC, intermediate care; SD, standard deviation; SQ, surprise question.

^*^ Statistically significant

Patients (mean age 83.2 years, 43% male) were mainly community dwelling. The mean Katz score and Lawton score were 8.4 (±3.5) and 4.6 (±2.1), respectively. More than half of the patients had two or more comorbidities (51.9%; *n* = 151), with a mean CCI of 5.9 (±2.3). Among the study patients, 46% (*n* = 135) required hospitalization, with 47% (*n* = 64) being admitted to a medicine ward and 28.9% (*n* = 39) to a geriatric ward.

Among patients with a positive SQ answered by EPs or ENs, it was observed that they were older and more often nursing home residents than those with a SQ−. They also displayed a higher functional dependency, based on the iADL and bADL scores. They took more medications per day and had a higher comorbidity score (CCI = 6.6 *versus* 5.4; *p* < 0.001). No significant difference was found between patients experiencing a fall within the past 12 months. Advance care planning was not available for the majority of the OP admitted (data not shown).

One-year mortality rate after ED visit was 21.6% and was significantly higher when EPs answered a positive SQ (SQ+ = 40% versus SQ− = 8%; *p* < 0.001).

### Prognostic accuracy of the surprise question and agreement between ED nurses and ED physicians

Table 3 displays the prognostic accuracy of the SQ when answered by EPs and ENs.

**Table 3.** Performance Tests of the Surprise Question

	Physicians			Nurses
	Any	Junior	Senior	
Sensitivity	0.79 (0.66–0.88)	0.7 (0.51–0.84)	0.91 (0.72–0.99)	0.71 (0.57–0.82)
Specificity	0.67 (0.6–0.73)	0.7 (0.63–0.78)	0.62 (0.5–0.73)	0.69 (0.62–0.75)
c-statistics	0.69 (0.63–0.75)	0.7 (0.62–0.77)	0.69 (0.58–0.78)	0.69 (0.63–0.75)
OR	7.33 (3.63–14.82)	5.49 (2.38–12.7)	16.88 (3.67–77.56)	5.39 (2.83–10.24)
PPV	0.4 (0.31–0.5)	0.39 (0.27–0.53)	0.43 (0.29–0.58)	0.41 (0.31–0.51)
NPV	0.92 (0.86–0.96)	0.9 (0.82–0.95)	0.96 (0.85–0.99)	0.89 (0.83–0.93)
PLR	2.36 (1.85–3)	2.36 (1.66–3.37)	2.38 (1.73–3.27)	2.29 (1.75–2.99)
NLR	0.32 (0.19–Z0.54)	0.43 (0.25–0.73)	0.14 (0.04–0.54)	0.42 (0.28–0.64)

NLR, negative likelihood ratio; NPV, negative predictive value; OR, odds ratio; PLR, positive likelihood ratio; PPV, positive predictive value.

Overall, accuracy in predicting 1-year mortality was moderate, whereas senior EPs showed a higher sensitivity and better NLR than junior EPs. In multivariable analysis adjusted for age, gender, and CCI, SQ + was associated with mortality with a hazard ratio (HR) of 3.2 [95%CI: 1.56—6.7] (*p* = 0.002).

The sensitivity was 0.79 (0.66–0.88) for EPs and 0.71 (0.57–0.82) for ENs, meaning that they correctly identified 79% and 71% of patients who would die within 12 months, respectively. EPs had a specificity of 0.67 (0.60–0.73), similar to that of ENs (0.69 [0.62–0.75]). The combined sensitivity, associating concordant responses between EPs and ENs, is lower (0.57) than that calculated separately. Conversely, the combined specificity is higher (0.85).

The probability of dying within 12 months in case of SQ+ (PLR) increased moderately for EPs (2.36 [1.85–3]) and ENs (2.29 [1.75–2.99]). The NLR was also moderate (EPs: 0.32 [0.19–0.54] and ENs: 0.42 [0.28–0.64]). When senior EPs responded SQ-, the informative value of the SQ− was higher (NLR 0.14 [0.04–0.54]).

The analyses of the SQ accuracy in predicting long-term mortality did not reveal any significant difference when comparing the responses of EPs and ENs (*p* = 0.67). The overall accuracy (c-statistics) of the SQ was 0.69 and remained consistent between EPs and ENs, with odds ratios of 7.33 (3.63–14.82) and 5.39 (2.83–10.24), respectively.

**FIG. 2.**
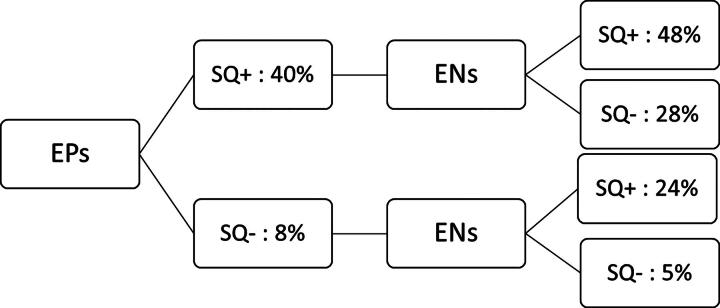
One-year mortality rates according the EPs’ responses, then combined with the ENs’ responses. EN, emergency nurses; EP, emergency physicians.

The mortality rate was higher when EPs or ENs answered “not surprised” (SQ+) (EPs: 39.3% (*n* = 48) *versus* 7.6% (*n* = 13), *p* < 0.001; ENs: 38.1% (*n* = 40) *versus* 11% (*n* = 17), *p* < 0.001). Survival rates at 1 year were found to be significantly lower when ENs or EPs provided a SQ + response (*p* < 0.001). Figure 2 shows the mortality rates at 1 year according to the EPs’ responses, then combined with the ENs’ responses.

**FIG. 3.**
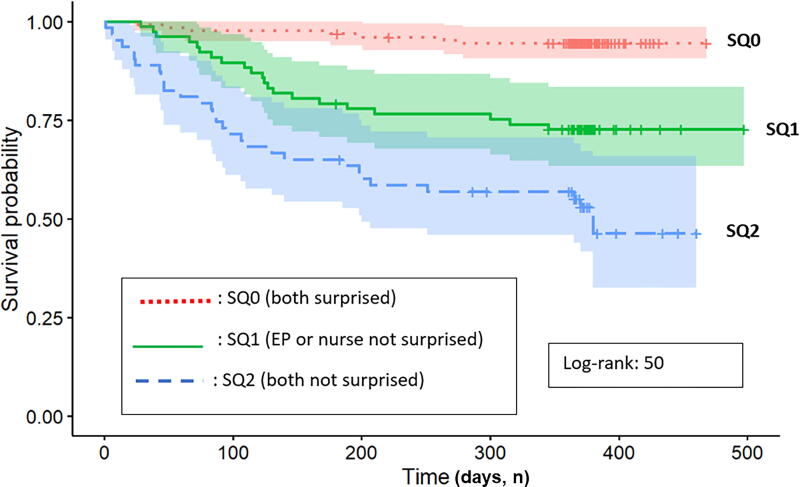
Survival differences among the SQ responses: “SQ0” when EPs and nurses answered “surprised,” “SQ1” when EPs OR nurses answered “not surprised,” and “SQ2” when both agreed on “not surprised.” EP, emergency physician; SQ, surprise question.

Interestingly, when both ENs and EPs answered a SQ+ (“SQ2”), survival curves show that OP die more rapidly compared with cases where only one of them was not surprised (“SQ1”) (log-rank 50; *p* < 0.001) (Fig. 3). These findings were statistically significant with a notable difference among the three survival curves (*p* < 0.001). Further analysis using Cox regressions, while adjusting for age, gender, and CCI, revealed that SQ + responses by both EPs and ENs were significantly associated with higher mortality rates, with HRs for EPs and ENs of 3.2 (97.5%CI: 1.56–6.7; *p* = 0.002) and 2.5 (97.5%CI: 1.30–4.8; *p* = 0.006), respectively.

EPs indicated that they would not be surprised (SQ+) if 43% (*n* = 113) of OPs admitted to the ED died within 12 months. When ENs answered, the SQ was positive for 40.6% of the patients (*n* = 106). There was an agreement between the two groups in approximatively 3 out of 4 patients (*n* = 171; 72.4%), regardless of whether the response was SQ- or SQ+ (Table 4). Both EPs and ENs agreed on a SQ + for more than 1 out of 4 patients (*n* = 65; 27.5%). The responses provided by EPs and ENs showed a significant association (*p* < 0.001) with a moderate level of agreement, as indicated by the Cohen’s kappa coefficient of 0.43 (0.31–0.56; *p* < 0.001).

**Table 4.** Agreement on the Surprise Question

	SQ + physicians, % (n)	SQ− physicians, % (n)
SQ + nurses, % (n)	27.5% (65)	11.9% (28)
SQ− nurses, % (n)	15.7% (37)	44.9% (106)

## Discussion

### Main findings

To the best of our knowledge, this study was the first to compare the prognostic value of the SQ between EPs and ENs in OPs within the ED setting. The accuracy of the SQ in predicting 1-year mortality was found to be moderate for both EPs and ENs. When both EPs and ENs independently agreed on an SQ+, patients had significantly a lower probability to be alive at one year, highlighting their complementarity in prognostication.

### Comparison to the literature

The accuracy of the SQ in this study aligned with previous research findings. Kirkland et al. (2022) highlighted that the SQ was the most widely screening tool used to identify unmet PC needs in the ED.^13^ The review included 12 studies on the SQ with different outcome measures (30-days, 6-month, and 12-month mortality). The sensitivity and specificity of the SQ to predict 12-month mortality varied widely across studies. Ouchi et al. investigated the prognostic accuracy of the SQ in older ED patients and reported similar results.^21^ Aaronson et al. found comparable performance measures in a cohort of heart failure patients admitted to the ED.^32^

The sensitivity of the SQ in this study was higher than that reported in other studies, especially for senior physicians.^33,34^ Higher sensitivity indicated that senior physicians were more successful in identifying patients who were likely to die within 1 year and who could potentially benefit from PC. On the contrary, the specificity of the SQ was lower than what has been reported in the literature.^13^ However, the NPV of the SQ was found to be high, indicating that a SQ− was reliable in ruling out patient at lower risk of 1-year mortality. Due to a lack of power, combined sensitivity was not statistically significant.

The higher sensitivity observed in this study could also be attributed to the severity of illness among the patients, resulting in an increased risk of death within 1 year. It should be noted that the mortality rate in this cohort was higher to findings from other studies conducted in EDs.^21^ The patients in our cohort were also older (inclusion criteria) and frailer than those in other studies where lower sensitivity was reported.^15,19^ Many factors related to the careworkers are reported in the literature to influence the prognosis accuracy of such clinical tool (specialty and relationship with the patients).^35^ EPs, who primarily encounter acutely ill patients in the ED, may tend to overestimate the mortality rate due to the perceived acuity of the cases they handle. On the contrary, physicians who have regular interactions with their patients could underestimate their mortality risk.^18,34^

The agreement between ENs and EPs in this study was found to be moderate, providing valuable insights as this is the first study to assess such agreement specifically in the ED setting. Yarnell et al.^33^ investigated the correlation between attending and trainee physicians in general internal medicine and observed a moderate agreement. In our study, we could not explore the reasons of disagreement on 28% of cases because of missing data. Another interesting question would be to see how predictions differed based on the patient’s survival.

### Clinical and research implications

The SQ can serve as a facilitator for end-of-life discussions and promotion of further assessment for potential unmet PC needs.^7^ The specific modalities for initiating a PC approach need to be clarified, as various studies have investigated different interventions, such as education of health care personnel, PC consultations, and automated screening tools.^36–38^

Although admission of OP in the ED is often seen as an undesirable event, the authors are convinced that it can be an opportunity to initiate a PC approach. This can benefit to the patient, their family but also in terms of health care services use. ED play a key role in the health care landscape, acting as a real hub that can also guide care trajectories. This role is particularly important when approaching the end of life. Indeed, three quarters of people who die have consulted an ED in their last 6 months of life, but few received PC.^8^ Although we have not tested the value of the answers of a “Cascading SQ,” agreement between ENs and EPs enables more specific identification of OP who will die in a year. This case finding raises a redflag to the professionals involved in the care of these patients, on the need to discuss the end-of-life issues and patients’ wishes. Moreover, this study underlines the importance of interprofessional collaboration and communication between EPs and ENs.

### Strengths and limits

This study has several strengths and limits. One of its strengths is its multicenter prospective design, with a cohort of OPs in an ED setting. This study also assessed, for the first time, the agreement between nurses and physicians.

We cannot generalize the results to an entire older population due to potential limits including selection bias (inclusion following the definition of the Belgian care program for the geriatric patient). We excluded patients admitted in the ED outside the office hours, dying patients (where it is too late to include them in an early palliative approach). We do not have any information about these patients’ characteristics. We also had to interrupt the inclusion during the COVID-19 pandemic.

The SQ was not initially built to predict death but to facilitate the identification of potential unmet PC needs.^12,13^ A number of studies have nevertheless evaluated this outcome.^17,18,21,23^

## Conclusion

The SQ is a simple tool to identify older ED patients at high mortality risk, even more when EPs and ENs independently agreed to be not surprised by a 1-year death. This could be a redflag to further assess OPs’ palliative care needs and initiate discussion about goals of care and end-of-life issues. Future interventional studies are needed to explore the impact of a “Cascading SQ”.

## Abbreviations Used

bADL: basic Activities of Daily Living
CCI: Charlson Comorbidity Index
ED(s): Emergency Department(s)
EMR: Electronic Medical Record
EN(s): Emergency Nurse(s)
EP(s): Emergency Physician(s)
HR: Hazard Ratio
iADL: instrumental Activities of Daily Living
ICU: Intensive Care Unit
IMC: Intermediate Care
NLR: Negative Likelihood Ratio
NPV: Negative Predictive Value
OP(s): Older Patient(s)
OR: Odds Ratio
PC: Palliative Care
PLR: Positive Likelihood Ratio
PPV: Positive Predictive Value
ROC: Receiver Operating Characteristics
SD: Standard Deviation
SQ: Surprise Question
